# Progranulin Protects Against Osteoporosis by Regulating Osteoclast and Osteoblast Balance via TNFR Pathway

**DOI:** 10.1111/jcmm.70385

**Published:** 2025-02-05

**Authors:** Shaoyi Wang, Hengyan Zhang, Yanbin Zhu, Xiaocong Zhou, Haoxin Zhai, Qiting He, Xuetao Zhu, Yuanqiang Zhang

**Affiliations:** ^1^ Department of Orthopaedic Surgery Qilu Hospital of Shandong University Jinan Shandong P. R. China; ^2^ Cheeloo College of Medicine Shandong University Jinan Shandong P. R. China; ^3^ Laboratory of Basic Medical Sciences Qilu Hospital, Cheeloo College of Medicine, Shandong University Jinan Shandong P. R. China; ^4^ The Second Children & Women's Healthcare of Jinan City Jinan Shandong P. R. China; ^5^ Department of Orthopaedic Surgery The Third Hospital of Hebei Medical University Shijiazhuang P. R. China; ^6^ Health Management Center The First Affiliated Hospital of Shandong First Medical University Jinan Shandong P. R. China

**Keywords:** osteoporosis, Progranulin, TNFR pathway

## Abstract

Osteoporosis is a disease of bone metabolism caused by an imbalance between osteoclast‐mediated bone destruction and osteoblast‐mediated bone formation. Tumour necrosis factor α (TNFα) has been reported to promote osteoclast generation and inhibit osteoblast generation. Progranulin (PGRN), which has a strong anti‐inflammatory effect, interacts with tumour necrosis factor receptor (TNFR). Serum and bone tissues from patients with or without osteoporosis were collected to analyse the relationship between PGRN content and bone metabolic markers. The role of TNFα and PGRN in osteoclast differentiation was explored by using RAW 264.7 cells and BMMs. MC3T3‐E1 cells and BMSCs were used to observe the role of TNFα and PGRN in osteoblast differentiation. The PGRN content in the serum and bone tissues of osteoporosis patients was lower than that in the serum and bone tissues of nonosteoporosis patients. TNFα promoted osteoclast differentiation, while PGRN inhibited this effect by interacting with TNFR1. PGRN inhibited TNFα‐mediated attenuation of osteoblast differentiation by interacting with TNFR1. Moreover, PGRN alone promoted osteoblast differentiation by interacting with TNFR2. Our findings reveal that PGRN can effectively inhibit TNFα‐induced osteoporosis and has a certain osteogenic effect. This discovery might provide a potential target for osteoporosis treatment.

**Abbreviations**
BMP2: bone morphogenetic protein‐2
Col‐1: Collagen 1
PGRN: progranulin
RUNX2: Runt‐related transcription factor 2
TNF‐α: Tumour Necrosis Factor α
T‐P1NP: Total type 1 collagen amino‐terminal prolongation peptide
TRAP: tartrate‐resistant acid phosphatase
VIT‐D: Vitamin D
β‐cross: β‐Collagen specific sequence

## 1 Introduction

Osteoporosis (OP) is a disease of bone metabolism caused by an imbalance between osteoclast‐mediated bone destruction and osteoblast‐mediated bone formation [1, 2, 3]. More than 50% of postmenopausal women and more than 30% of older men are reported to have osteoporotic fractures, and the incidence is increasing with increasing longevity [4]. Osteoporosis has a high incidence rate and easily leads to serious consequences, such as fracture, which greatly increases the economic and mental burden on the family and society [5].

Recent studies have shown that inflammation is closely related to the development of osteoporosis [6, 7, 8]. Tumour necrosis factor α (TNFα) is closely associated with inflammation and has been reported to promote osteoclast generation and inhibit osteoblast generation [9, 10]. It has been reported that TNFα promotes osteoclast differentiation through the ODF(Osteoclast differentiation factor)/RANKL(receptor activator of nuclear factor‐κB ligand)‐RANK signalling pathway [11]. In addition, TNFα has also been reported to inhibit osteoblast formation through the β‐catenin pathway [12].

Progranulin is a 593 amino acid autocrine growth factor that plays a critical role in many pathological and physiological processes, such as inflammation, host defence, wound healing and intervertebral disc degeneration [13, 14, 15, 16, 17, 18, 19, 20, 21, 22, 23, 24, 25]. PGRN exerts its diverse functions through interactions with numerous associated proteins [26]. Remarkably, it demonstrates the ability to bind to a broad spectrum of proteins across various cellular contexts, including the extracellular environment, the extracellular matrix (ECM), and intracellular compartments, such as ADAMTS7, ADAMTS12, COMP, GCase and Sortilin [27, 28, 29, 30]. Notably, PGRN was reported to interact with tumour necrosis factor receptors (TNFR) [31], hence inhibiting TNFα's proinflammatory effects. In osteoarthritis and atherosclerosis, PGRN exerts its anti‐inflammatory effects through interaction with TNFR [16, 32]. By interacting with TNFR1, PGRN inhibits the progression of inflammation in patients with type 2 diabetes and chronic periodontitis [33, 34]. In addition to suppressing TNFα‐induced inflammatory responses, PGRN has also been reported to exhibit anti‐inflammatory effects under conditions of inflammation induced by interleukin‐1β (IL‐1β) and lipopolysaccharides (LPS) [35]. Furthermore, recent studies demonstrated that PGRN plays critical roles in bone metabolism. Zhao et al. found that titanium particles could cause skull osteolysis in mice by activating the NF‐κB pathway and that PGRN could attenuate this effect to prevent osteolysis [36]. Furthermore, Noguchi et al. used PGRN knockout mice to prove that loss of PGRN can lead to bone loss when compared with the control littermates [37]. Thus, we hypothesized that PGRN plays an important role in osteoporosis.

In this study, we compared PGRN levels in serum and bone tissue in patients with and without osteoporosis. Then we used RAW264.7 cells and bone marrow‐derived macrophages (BMMs) to induce osteoclast differentiation, MC3T3‐E1 cells and bone marrow mesenchymal stem cells (BMSCs) to induce osteoblast differentiation. We then determined whether PGRN is associated with osteoporosis and investigated the role of PGRN in osteoporosis.

## 2 Materials and Methods

### 2.1 Patients and Samples

Clinical sample inclusion criteria: (1), Patients undergoing single stage posterior lumbar fusion surgery in our hospital for lumbar disc herniation alone. (2), No autoimmune disease, cardiovascular disease and metabolic disease. (3), The patient had no history of haematological infectious diseases in the past 6 months. (4), Patients did not take long‐term (continuous use greater than 1 year) drugs that affect bone metabolism, such as glucocorticoids. According to whether the patient has osteoporosis clinical sample divided into CON group and OP group (whether the T‐value of hip BMD measured by dual‐energy X‐ray was less than −2.5. Less than −2.5 was OP group). Finally, the number of patients enrolled in CON group was 20, and the number of patients in the OP group was 25. The basic characteristics between the two groups were shown in Table 1.

**TABLE 1:** Basic characteristics of the research object.

	CON group (*n* = 20)	OP group (*n* = 25)	*p*
Age (years)	61.3 ± 1.197	63.88 ± 0.8373	0.2543
Sex	Males (*n* = 20)	Males (*n* = 25)	
BMI (kg/m^2^)	24.15 ± 0.577	23.72 ± 0.4883	0.7875

Blood samples were taken before surgery on an empty stomach. No patient had fever or other acute disease during blood collection. We extracted four millilitres of blood from each patient into a heparin anticoagulation tube in the morning. Then, blood samples were sent to the laboratory, centrifuged at 4°C at 2000 × *g* for 15 min and then stored at −20°C for testing.

**TABLE 2:** Real‐Time PCR primers.

Target	Forward primers,5′‐3′	Reverse primers,5′‐3′
TRAP	AGCAGCCAAGGAGGACTAC	CATAGCCCACACCGTTCTC
Cathepsin K	AGCTTCCCCAAGATGTGAT	AGCACCAACGAGAGGAGAA
Caltonin receptor	CTCCTTGTCGATTGCTGCT	TCACCCTCTGGCAGCTAAG
β‐Actin	CCTCATGAAGATCCTGACCG	ACCGCTCATTGCCGATAGTG
Col‐1	GACATGTTCAGCTTTGTGGACCTC	AGGGACCCTTAGGCCATTGTGTA
RUNX2	TCTGACAAAGCCTTCATGTCC	AAATAGTGATACCGTAGATGCG
scRNAi	UUCUCCGAACGUGUCACGUTT	ACGUGACACGUUCGGAGAATT
TNFR1 RNAi1	CCGCUUGCAAAUGUCACAATT	UUGUGACAUUUGCAAGCGGTT
TNFR1 RNAi2	CCGAAGUCUACUCCAUCAUTT	AUGAUGGAGUAGACUUCGGTT
TNFR1 RNAi3	GGAGAUCUCUCCUUGCCAATT	UUGGCAAGGAGAGAUCUCCTT
TNFR2 RNAi1	CUGGCCAAUAUGUGAAACATT	UGUUUCACAUAUUGGCCAGTT
TNFR2 RNAi2	CUGGCUGGUUUGAUCAGAUTT	AUCUGAUCAAACCAGCCAGTT
TNFR2 RNAi3	CCAAACUCCAAGCAUCCUUTT	AAGGAUGCUUGGAGUUUGGTT

Bone tissues were collected from 6 patients in OP group. The target vertebral body T value was less than −2.5. The CON group bone tissues underwent the same surgery but the target vertebral body T value was more than −1. Bone tissue was rinsed with sterile saline and stored at −80°C until testing. Sequence‐specific primers for PGRN are listed in Table 2.

All patients involved in the study signed informed consent forms, and the study was approved by the medical ethics regulations of the Medical Ethical Committee of Qilu Hospital of Shandong University.

### 2.2 Mouse Bone Marrow‐Derived Macrophages

Mouse bone marrow‐derived macrophages (BMMs) was obtained from 12‐week‐old female mice (C57BL/6, Shandong University, China) by flushing the bone marrow cavities of both femur and tibia. After 24 h, the rinse solution is absorbed and centrifuged. The precipitated cells were transferred to a new culture flask and cultured with Dulbecco's modified Eagle's medium (DMEM) (Gibco BRL, MD) containing 10% fetal bovine serum (FBS), 100 U/mL penicillin and 0.1 mg/mL streptomycin (HyClone, USA) under standard conditions (37°C, 5% CO_2_). After that, the medium was replaced every 3 days.

### 2.3 Mouse Bone Marrow Mesenchymal Stem Cells

Mouse Bone Marrow Mesenchymal Stem Cells was obtained from 12‐week‐old female mice (C57BL/6, Shandong University, China) by flushing the bone marrow cavities of both femur and tibia. Discard the rinse solution after 24 h, and cells were cultured with minimum essential medium α (MEMα) (Gibco BRL, MD) containing 10% fetal bovine serum (FBS), 100 U/mL penicillin and 0.1 mg/mL streptomycin (HyClone, USA) under standard conditions (37°C, 5% CO_2_). After that, the medium was replaced every 3 days.

### 2.4 Cell Culture

RAW 264.7 cells were maintained in Dulbecco's modified Eagle's medium (DMEM) (Gibco BRL, MD) containing 10% fetal bovine serum (FBS), 100 U/mL penicillin and 0.1 mg/mL streptomycin (HyClone, USA) and incubated under standard conditions (37°C, 5% CO_2_).

MC3T3‐E1 cells were maintained in minimum essential medium α (MEMα) (Gibco BRL, MD) containing 10% fetal bovine serum (FBS), 100 U/mL penicillin and 0.1 mg/mL streptomycin (HyClone, USA) and incubated under standard conditions (37°C, 5% CO_2_).

### 2.5 In Vitro Osteoclast Differentiation

To induce osteoclast differentiation, RAW 264.7 cells and BMMs were stimulated with 50 ng/mL receptor activator of nuclear factor kappa‐B ligand (RANKL, Peprotech, USA) and 100 ng/mL macrophage colony‐stimulating factor (M‐CSF, Peprotecch, USA) [38]. To test whether TNFα can enhance RANKL‐mediated osteoclast differentiation, RAW 264.7 cells and BMMs were cultured with 10 ng/mL TNFα (Peprotech, USA). To test whether PGRN can suppress TNFα‐mediated osteoclast differentiation, RAW 264.7 cells and BMMs were cultured with 10 ng/mL TNFα with or without 500 ng/mL rhPGRN (R&D Systems, USA) [39]. Cells were harvested and assessed at 8 h by real‐time PCR, at 3 days by Western blotting and at 7 days by TRAP staining.

### 2.6 In Vitro Osteoblast Differentiation

To induce osteoblast differentiation, MC3T3‐E1 cells and BMSCs were stimulated with 10 mM β‐glycerophosphate and 50 μg/mL ascorbate phosphate [40]. To test whether TNFα can suppress osteoblast differentiation, MC3T3‐E1 cells and BMSCs were cultured with 10 ng/mL TNFα (Peprotech, USA). To test the role of PGRN in osteoblast differentiation, MC3T3‐E1 cells and BMSCs were cultured with 10 ng/mL TNFα and 500 ng/mL rhPGRN (R&D Systems, USA). rhPGRN (500 ng/mL) was used to determine whether PGRN could promote osteoblast differentiation. Cells were harvested and tested at 8 h by real‐time PCR, at 3 days by Western blotting, at 14 days by alkaline phosphatase staining and at 21 days by alizarin red staining.

### 2.7 Co‐Immunoprecipitation (CO‐IP)

Proteins were extracted from BMMs and MC3T3‐E1 cells after incubated with or without rhPGRN for 1 h. The cells were lysed in RIPA buffer (Millipore, Billerica, MA, USA) supplemented with 5% PMSF, a protease inhibitor, and kept on ice for 40 min. The lysates were centrifuged at 12,000 rpm for 15 min at 4°C, and the resulting supernatant was collected. To prevent non‐specific protein interactions, 4 μL of Protein A/G PLUS‐agarose beads (Santa Cruz, USA) were combined with 200 μL of pre‐cleared lysates and 0.4 μL of immunoglobulin (IgG) from the same host species as the primary antibody (rabbit anti‐goat IgG, Zsbio, China) and incubated at 4°C for 1 h. After centrifugation under the same conditions, the supernatant was retained. Protein concentration was measured using a BCA protein assay kit (Biotechnology Co, Beijing, China), following the manufacturer's guidelines, and adjusted to 1 μg/μL. For immunoprecipitation, 200 μL of the adjusted lysate and 8 μL of Protein A/G PLUS‐agarose beads were incubated overnight with 2 μL of primary antibody (rabbit anti‐PGRN, 1:200, Abcam, USA) or (rabbit anti‐TNFα, 1:200, Abcam, USA) on ice with gentle shaking. The next day, the beads were washed three times with cold PBS, and the bound proteins were eluted with 50 μL of SDS‐PAGE sample loading buffer (Beyotime, China) by heating at 95°C for 10 min. The eluted proteins were then subjected to Western blot analysis.

### 2.8 Total Protein Extraction and Western Blotting

The cells were washed with sterile phosphate buffer (PBS) 3 times, and bone tissue powder was placed in RIPA lysis buffer (Millipore, Billerica, MA, USA) supplemented with 5% PMSF (a protease inhibitor) and 1% protein phosphatase inhibitor (Solarbio, China) on ice for 30 min. Then, the lysates were centrifuged at 12,000 r/m for 15 min at 4°C. The supernatant was retained. The protein concentration was measured using a BCA protein assay kit (Biotechnology Co, Beijing, China). Equal amounts of protein from each group were separated in 10% SDS–polyacrylamide gels (SDS–PAGE) and transferred to a polyvinylidene difluoride (PVDF) membrane (Millipore, USA). PVDF membranes were blocked in tris‐buffered saline Tween‐20 (TBST) with 5% skimmed milk powder for 1 h and incubated with rabbit‐anti‐GAPDH (1:5000, Proteintech, USA), rabbit anti‐PGRN (1: 1500, Abcam, USA), rabbit‐anti‐TRAP (1:500, Abcam, USA), rabbit‐anti‐RUNX2 (1:1000, Abcam, USA), rabbit‐anti‐Col‐1 (1:1000, Abcam, USA), rabbit‐anti‐p‐P65(1:1000, CST, USA), rabbit‐anti‐P65(1:1000, CST, USA), rabbit‐anti‐p‐Erk1/2 (1:1000, CST, USA), rabbit‐anti‐Erk1/2 (1:1000, CST, USA), rabbit‐anti‐p‐IkBα (1:1000, CST, USA), rabbit‐anti‐IkBα (1:1000, CST, USA), rabbit‐anti‐p‐JNK (1:1000, CST, USA), rabbit‐anti‐JNK (1:1000, CST, USA), rabbit‐anti‐TNFR1(1:1000, Affinity, USA) and rabbit‐anti‐TNFR2(11,000, Affinity, USA) antibodies overnight at 4°C. On the second day, the membrane was washed 3 times with TBST for 10 min and incubated with a goat anti‐rabbit immunoglobulin (IgG)‐horseradish peroxidase (HRP) secondary antibody (15,000, Jackson ImmunoResearch, USA) at room temperature for 1 h. A FluorChem E Chemiluminescent Western Blot Imaging System (Amersham Imager 600, GE Amersham USA) was used to visualise the protein bands, which were quantified by densitometry analysis using ImageJ software (National Institutes of Health, USA). The differences between the groups were statistically analysed with the measured grey values.

### 2.9 Cell Treatment

To further investigate the role of the NF‐κB signalling pathway, NF‐κB inhibitor BAY11‐7082 (5 μM) [41] was added to BMMs and MC3T3‐E1 cells with or without rhPGRN and TNFα during osteoclast differentiation. Cells were harvested and tested a *t* 8 h by real‐time PCR, at 3 days by Western blotting.

To further investigate the involvement of the ERK signalling pathway, ERK inhibitor SCH772984 (5 μM) (MCE, USA) [42] was added to MC3T3‐E1 cells with or without rhPGRN during osteoblast differentiation. Cells were harvested and tested at 8 h by real‐time PCR, at 3 days by Western blotting.

### 2.10 Statistical Analyses

Statistics were analysed by GraphPad Prism 7.1 (GraphPad Software Inc., San Diego, CA, USA). Data are expressed as the mean value ± standard deviation (SD). Significance was assessed using a one‐way analysis of variance (ANOVA) or *T* test, and *p* < 0.05 was considered significant. The correlation between the concentration of PGRN and bone metabolism markers was analysed by Spearman's correlation coefficient.

More detailed experimental procedures are described in the online Data S1: Materials and Methods.

## 3 Results

### 3.1 PGRN Was Reduced in the Peripheral Blood and Associated With Markers of Serum Bone Metabolism in Patients With Osteoporosis

**FIGURE 1:**
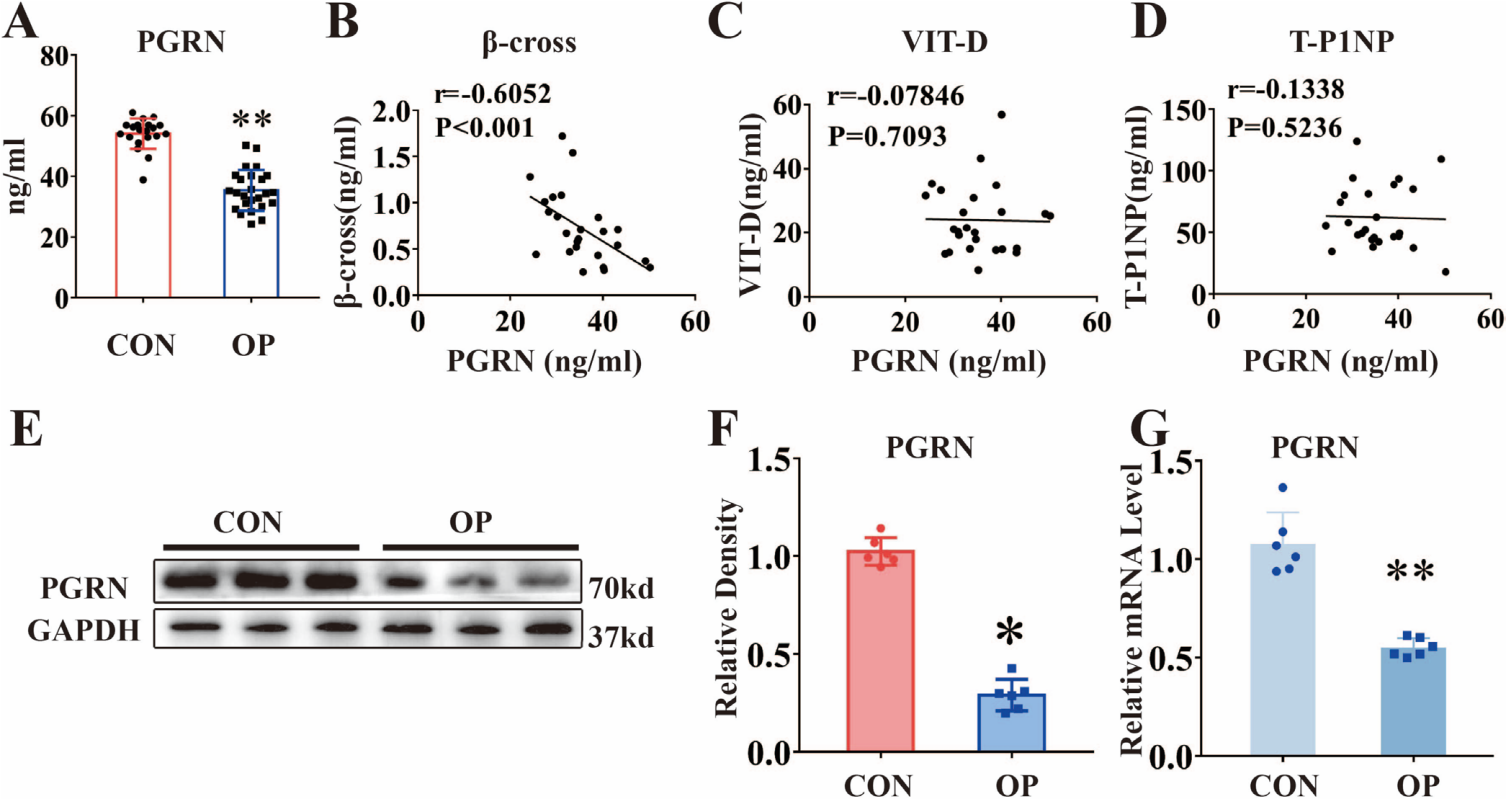
PGRN was associated with markers of serum bone metabolism in patients with osteoporosis and reduced in the peripheral blood and bone tissues of patients with OP. (A) The content of PGRN in serum was detected by ELISA. The serum PGRN content of patients without osteoporosis was 54.08 ± 1.122 ng/mL. The serum PGRN content of patients with osteoporosis was 35.4 ± 1.347 ng/mL. (B) The Spearman's correlation coefficients were negative for the correlation of the serum level of β‐cross and the serum level of PGRN in patients with osteoporosis (*r* = −0.6052, *p* < 0.001). (C) The Spearman's correlation showed that there was no statistically significant correlation between serum level of VIT‐D and the serum level of PGRN in patients with osteoporosis (*r* = −0.07846, *p* = 0.7093). (D) The Spearman's correlation showed that there was no statistically significant correlation between serum level of T‐P1NP and the serum level of PGRN in patients with osteoporosis (*r* = −0.1338, *p* = 0.5236). (E) Western blot (WB) analysis of PGRN in bone tissues from the patients with or without osteoporosis. (F) Quantification of WB analysis (*n* = 6 for each group). (G) Real‐time PCR of PGRN in bone tissues of the indicated groups (*n* = 6 for each group). Data were presented as the mean ± SD. *p < 0.05, **p < 0.01.

First, serum PGRN levels in both nonosteoporotic and osteoporotic patients were measured. As shown in Figure 1A, the content of PGRN in the serum of nonosteoporosis patients was 54.08 ± 1.122 ng/mL, which was higher than that of osteoporosis patients (35.4 ± 1.347 ng/mL). To investigate the relationship between serum PGRN content and bone metabolism markers in osteoporosis patients, we analysed the relationship between PGRN and three markers of bone metabolism. As shown in Figure 1B, with the increase in serum PGRN content, the level of β‐cross decreased. Spearman's correlation coefficient R was −0.6052, which showed a relatively strong relationship. As illustrated in Figure 1C, there was no correlation between VIT‐D and PGRN serum content. Similarly, as shown in Figure 1D, there is no correlation between T‐P1NP and PGRN. Subsequently, we examined the changes in PGRN content in vertebral bone tissues. As illustrated in Figure 1E,F, the protein level of PGRN in bone tissues of osteoporosis patients was decreased. In addition, compared with the patients without osteoporosis, the transcriptional level of PGRN in the bone tissues of osteoporosis patients was significantly decreased in OP patients (Figure 1G).

### 3.2 PGRN Suppressed TNFα‐Mediated Inflammatory Osteoclast Differentiation by Suppressing the Activation of the NF‐κB Pathway Induced by TNFα

**FIGURE 2:**
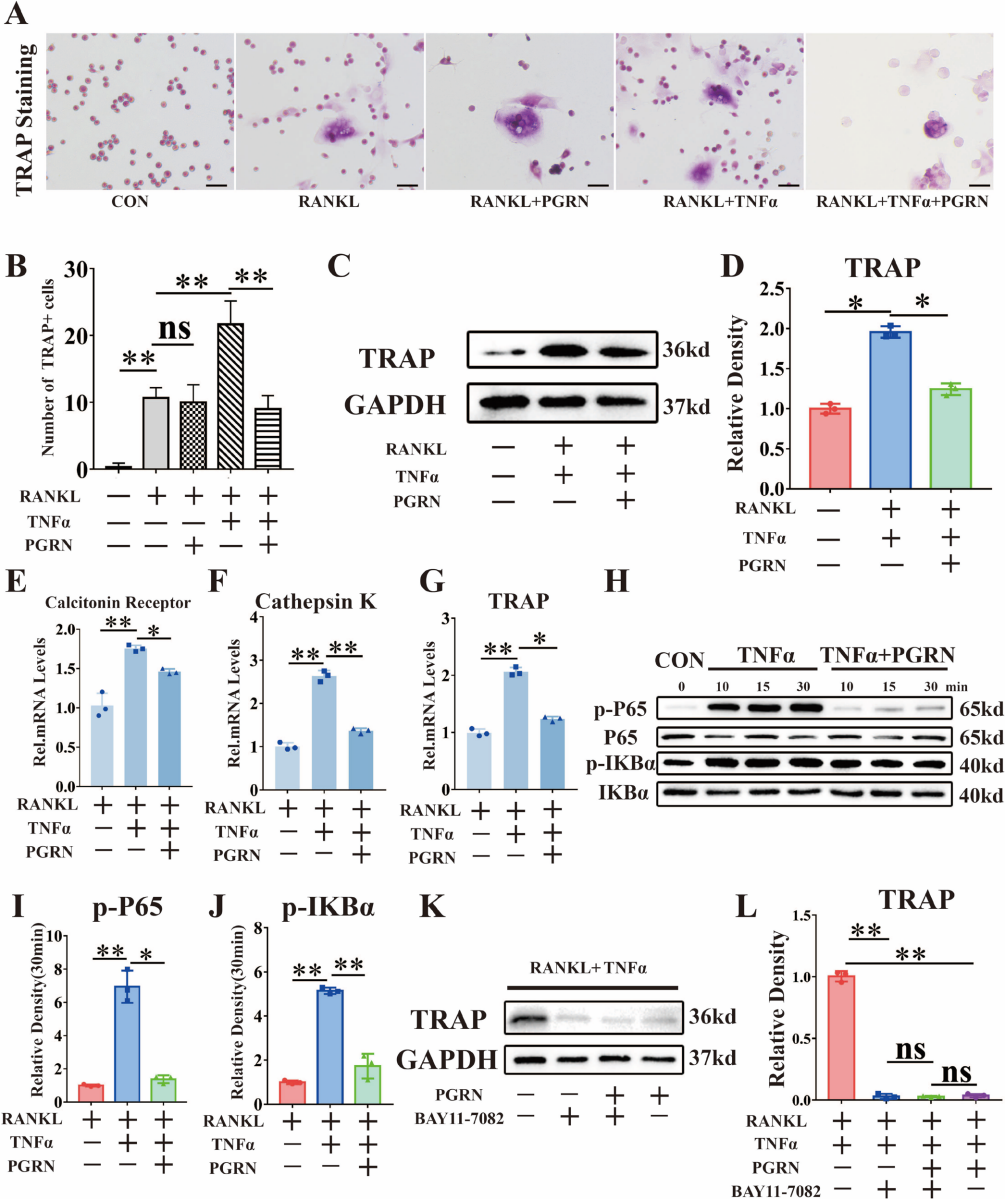
PGRN suppressed TNFα‐mediated inflammatory osteoclast differentiation by suppressing the activation of the NF‐κB pathway induced by TNFα. (A) Representative images of TRAP staining of BMMs after incubated with TNFα and PGRN for 7 days. Scale bars, 100 μm. (B) Quantification of TRAP staining was analysed by number of TRAP+ cells (*n* = 3 for each group). (C) Western blot (WB) analysis of TRAP of BMMs after incubated with TNFα and PGRN for 3 days. (D) Quantification of WB analysis (*n* = 3 for each group). (E–G) Real‐time PCR of Calcitonin Receptor, Cathepsin K and TRAP after addition of TNFα and PGRN for 8 h (*n* = 3 for each group). (H) Western blot (WB) analysis of p‐P65, P65, p‐IKBα, IKBα of BMMs cultured with TNFα and PGRN. (I, J) Quantification of WB analysis (*n* = 3 for each group). (K) WB analysis of TRAP of BMMs after incubated with TNFα, PGRN and BAY11‐7082(NF‐κB inhibitor) for 3 days. (L) Quantification of WB analysis (*n* = 3 for each group). Concentration: TNFα (10 ng/mL), rhPGRN (500 ng/mL), BAY11‐7082 (5 μM). Data were presented as the mean ± SD. *p < 0.05, **p < 0.01.

We cultured BMMs and RAW 264.7 cells, and induced in vitro osteoclast differentiation with RANKL and TNFα. First, TRAP staining of BMMs showed that TNFα enhanced osteoclast differentiation, while PGRN inhibited this effect (Figure 2A,B). After 3 days of stimulation, we extracted total cellular protein from BMMs and RAW 264.7 cells. As shown in Figure 2C,D and Figure S1A,B, TNFα upregulated the protein production of TRAP, while PGRN inhibited this effect. Real‐time PCR for osteoclastic biomarkers, including TRAP, calcitonin receptor and cathepsin K, was performed. The results showed that TNFα could promote the expression of osteoclast differentiation markers. PGRN reduced the promoting effect of TNFα (Figure 2E–G, Figure S1C–E). Moreover, We further investigated the molecular mechanisms of TNFα and PGRN in osteogenic and osteoclast differentiation. During osteoclast differentiation, TNFα enhanced inflammatory osteocla ***s*** t differentiation by promoting phosphorylation of the P65, IKBα proteins. And PGRN reduced the phosphorylation of these proteins (Figure 2H–J). Finally, we added the NF‐κB inhibitor, and the results demonstrated that the activation of osteoclasts induced by TNFα could be effectively suppressed by the NF‐κB inhibitor, showing a similar effect to that of PGRN. Furthermore, the addition of the NF‐κB inhibitor to PGRN did not further reduce the level of osteoclast activation (Figure 2K,L).

### 3.3 PGRN Reduced the Inhibitory Effect of TNFα on Osteoblast Differentiation by Suppressing the Activation of the NF‐κB Pathway Induced by TNFα

**FIGURE 3:**
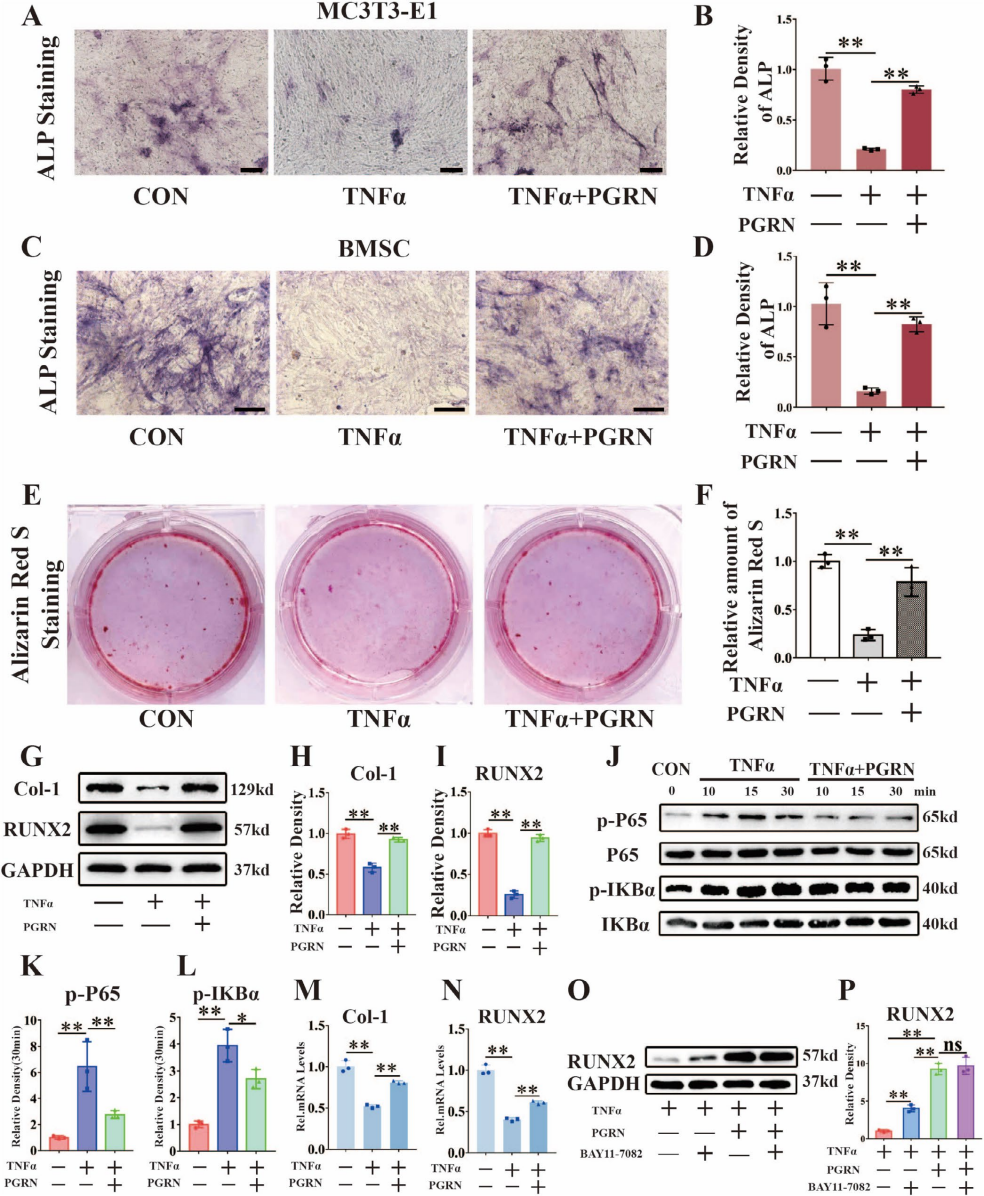
PGRN reduced the inhibitory effect of TNFα on osteoblast differentiation by suppressing the activation of the NF‐κB pathway induced by TNFα. (A) Representative images of ALP staining of MC3T3‐E1 cells co‐cultured with TNFα and PGRN for osteogenic induction for 14 days. Scale bars, 100 μm. (B) Quantification of ALP staining analysis (*n* = 3 for each group). (C) Representative images of ALP staining of BMSCs co‐cultured with TNFα and PGRN for osteogenic induction for 14 days. Scale bars, 100 μm. (D) Quantification of ALP staining analysis (*n* = 3 for each group). (E) Representative images of Alizarin Red S staining of MC3T3‐E1 cells co‐cultured with TNFα and PGRN for osteogenic induction for 21 days. (F) Quantitative analysis of Alizarin Red S staining was detected by relative amounts of Alizarin Red S (*n* = 3 for each group). (G) Western blot (WB) analysis of Col‐1 and RUNX2 after incubated with TNFα and PGRN for 3 days. (H, I) Quantification of WB analysis (*n* = 3 for each group). (J) WB analysis of p‐P65, P65, p‐IKBα and IKBα in MC3T3‐E1 cells cultured with TNFα and PGRN. (K, L). Quantification of WB analysis (*n* = 3 for each group). (M, N) Real‐time PCR analysis of Col‐1 and RUNX2 (*n* = 3 for each group) after stimulated for 8 h. (O) WB analysis of TRAP of MC3T3‐E1 after incubated with TNFα, PGRN and BAY11‐7082(NF‐κB inhibitor) for 3 days. (P) Quantification of WB analysis (*n* = 3 for each group). Concentration: TNFα (10 ng/mL), rhPGRN (500 ng/mL), BAY11‐7082 (5 μM). Data were presented as the mean ± SD. *p < 0.05, **p < 0.01.

To verify the role of PGRN and TNFα in osteoblast differentiation, we cultured MC3T3‐E1 cells and BMSCs to induce osteogenic differentiation in vitro. First, ALP staining was performed. The activity of ALP was decreased significantly after incubation with TNFα. However, PGRN reversed the activity of ALP (Figure 3A–D). To assess matrix mineralization, alizarin red S staining was performed. TNFα significantly inhibited the production of calcium nodules. PGRN restored the number of calcium nodules (Figure 3E,F). After 3 days of osteoblast induction in vitro, total protein was extracted for WB analysis. We examined two osteogenic markers, runt‐related transcription factor 2 (RUNX2) and collagen 1 (Col‐1). As indicated in Figure 3G–I, TNFα significantly inhibited the expression of these two osteogenic markers. However, after incubation with PGRN, the protein production of these two osteogenic markers increased. Moreover, during osteoblast differentiation, TNFα promoted the phosphorylation of the P65 and IKBα proteins, while PGRN inhibited their phosphorylation (Figure 3J–L). Real‐time PCR for osteoblastic biomarkers, including RUNX2 and Col‐1, was performed (Figure 3M,N). The results showed that TNFα downregulated the expression of these markers and that PGRN inhibited the TNFα effect. During osteoblast differentiation, we added NF‐κB inhibitor. As showed in Figure 3O,P, the addition of an NF‐κB inhibitor restored osteogenesis, while treatment with PGRN further enhanced osteoblast activation. Notably, there was no significant difference in osteoblast activation levels between groups treated with PGRN alone and those treated with both PGRN and the NF‐κB inhibitor. These findings suggest that PGRN not only counteracts TNFα‐mediated inhibition of osteogenesis but may also promote osteoblast activation through additional pathways.

### 3.4 PGRN Suppressed TNFα‐Mediated Inflammatory Osteoclast Differentiation by Interacting With TNFR1

**FIGURE 4:**
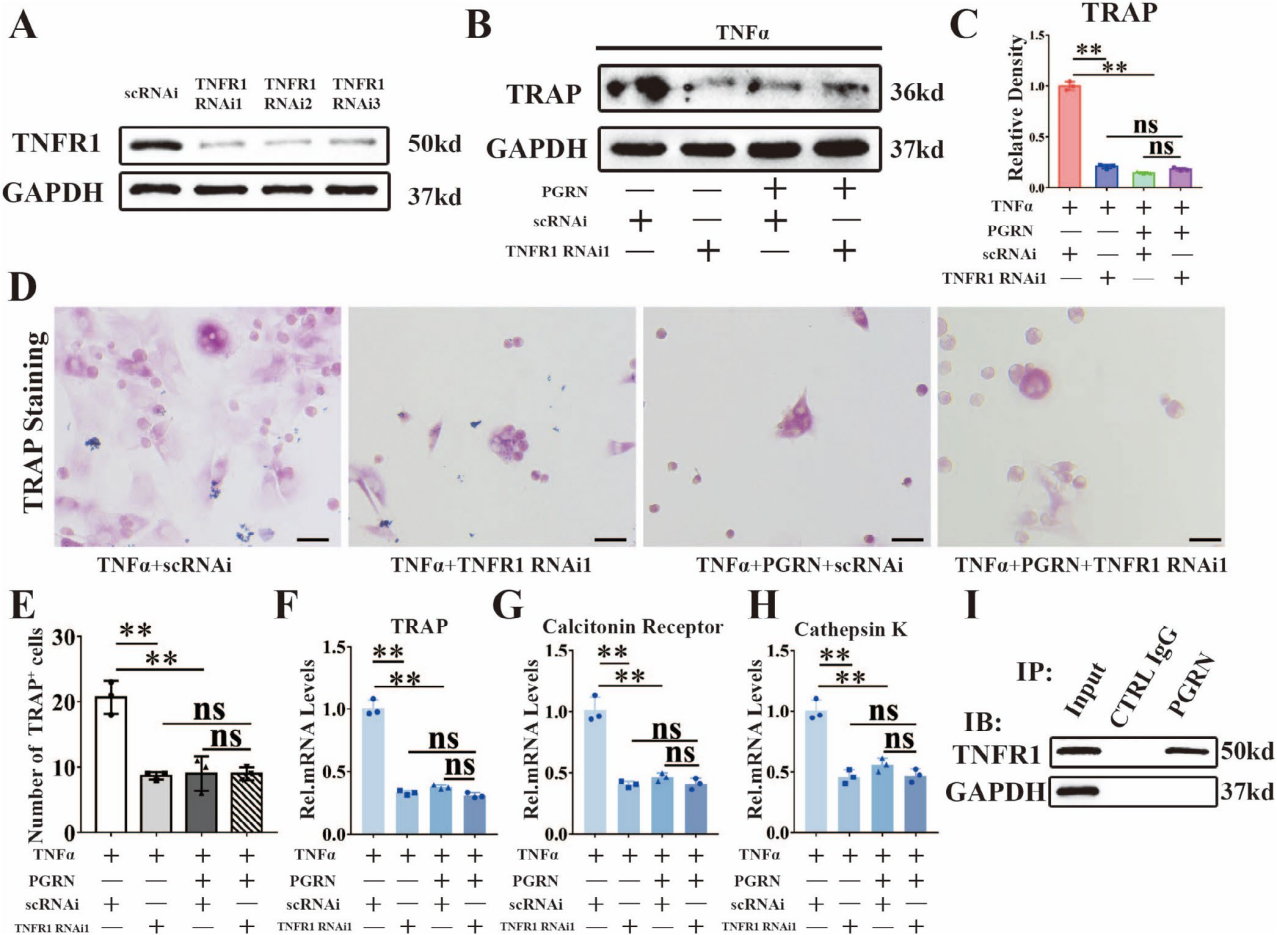
PGRN suppressed TNFα‐mediated inflammatory osteoclast differentiation by interacting with TNFR1. (A) Western blot (WB) analysis of TNFR1 to detect the knockdown efficacy of TNFR1 RNAi in BMMs after cultured for 3 days. (B) WB analysis of TRAP in BMMs cultured with TNFα, PGRN and TNFR1 RNAi for 3 days. (C) Quantification of WB analysis (*n* = 3 for each group). (D) Representative images of TRAP staining of BMMs after incubated with TNFα, PGRN and TNFR1 RNAi for 7 days. Scale bars, 50 μm. (E) Quantification of TRAP staining was analysed by number of TRAP+ cells (*n* = 3 for each group). (F–H) Real‐time PCR of Calcitonin Receptor, Cathepsin K and TRAP of the indicated groups (*n* = 3 for each group) after stimulated for 8 h. (I) Immunoprecipitation (IP) with anti‐PGRN or control immunoglobulin G (IgG) followed by Western blotting for TNFR1 (*n* = 3). IB: Immunoblot. Concentration: TNFα (10 ng/mL), rhPGRN (500 ng/mL). Data were presented as the mean ± SD. **p < 0.01.

TNFα plays a role primarily by interacting with the TNF receptors TNFR1 and TNFR2 [43]. Current studies suggest that TNFR1 plays an inflammatory role, while TNFR2 plays a protective role [23]. Next, we examined whether PGRN inhibited TNFα‐mediated osteoclast differentiation through TNFR1 or TNFR2. We used specific siRNAs to inhibit TNFR1 expression in BMMs. TNFR1 expression was detected 3 days after stimulation. As shown in Figure 4A, the protein production of TNFR1 was significantly inhibited. Osteoclast differentiation was performed for 3 days after knocking down the expression of TNFR1, the promoting effect of TNFα on TRAP was weakened, and the level of TRAP did not decrease further after PGRN was added (Figure 4B,C). After knocking down TNFR1, PGRN could not play the role of inhibiting TNFα. TRAP staining of BMMs showed that the number of TRAP^+^ cells decreased significantly after TNFR1 knockdown but showed no significant change after PGRN addition (Figure 4D,E). Real‐time PCR of TRAP, calcitonin receptor and cathepsin K confirmed the previous findings (Figure 4F–H). Co‐immunoprecipitation (Co‐IP) experiments confirmed the interaction between PGRN and TNFR1 during osteoclast activation (Figure 4I). Furthermore, TNFR1 neutralising antibody was added into the osteoclast differentiation of BMMs. WB of TRAP (Figure S2A,B) and Real‐time PCR (Figure S2C–E) results showed TNFα promoted TRAP expression dependent on TNFR1, and PGRN also lost its effect on TNFα‐mediated osteoclast differentiation after neutralising TNFR1. During osteoclast activation, the addition of PGRN significantly reduced the interaction strength between TNFα and TNFR1 (Figure S3A,B). In summary, PGRN suppressed TNFα‐induced osteoclast differentiation by interacting with TNFR1.

### 3.5 PGRN Reduced the Inhibitory Effect of TNFα on Osteoblast Differentiation by Interacting With TNFR1

**FIGURE 5:**
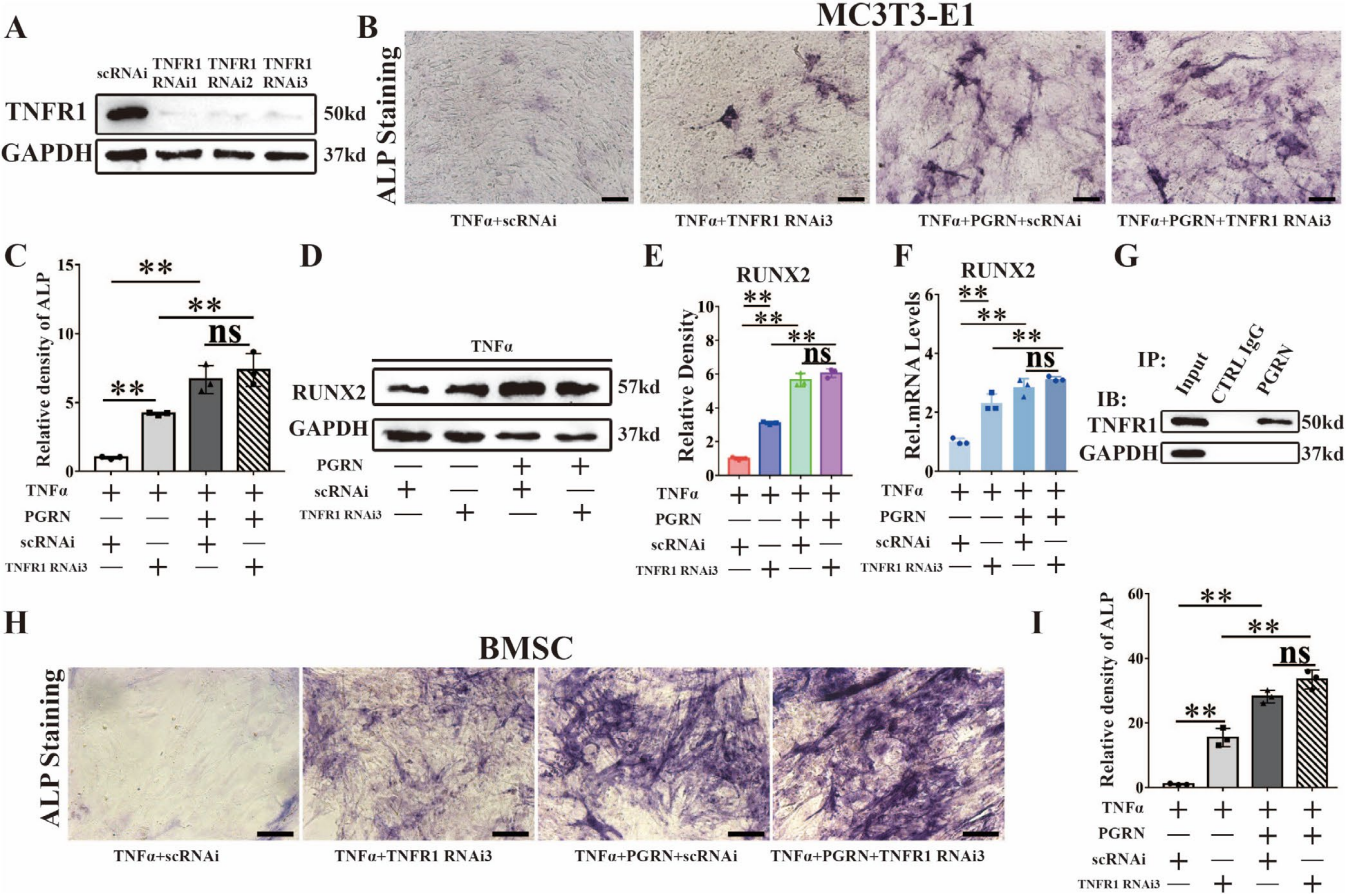
PGRN reduced the inhibitory effect of TNFα on osteoblast differentiation by interacting with TNFR1. (A) Western blot (WB) analysis of TNFR1 to detect the knockdown efficacy of TNFR1 RNAi in MC3T3‐E1 cells for 3 days. (B) Representative images of ALP staining of MC3T3‐E1 cells co‐cultured with TNFα, PGRN and TNFR1 RNAi for osteogenic induction for 14 days. Scale bars, 100 μm. (C) Quantification of ALP staining analysis (*n* = 3 for each group). (D) Western blot analysis of RUNX2 of the indicated groups after cultured for 3 days. (E) Quantification of WB analysis (*n* = 3 for each group). (F) Real‐time PCR of RUNX2 of the indicated groups (*n* = 3 for each group). (G) Immunoprecipitation (IP) with anti‐PGRN or control IgG followed by Western blotting for TNFR1 (*n* = 3). IB: Immunoblot. (H) Representative images of ALP staining of BMSCs co‐cultured with TNFα, PGRN and TNFR1 RNAi for osteogenic induction for 14 days. Scale bars, 100 μm. (I) Quantification of ALP staining analysis (*n* = 3 for each group). Concentration: TNFα (10 ng/mL), rhPGRN (500 ng/mL). Data were presented as the mean ± SD. **p < 0.01.

During osteoblast differentiation, we used specific siRNAs and TNFR1 neutralising antibody to inhibit TNFR1 expression in MC3T3‐E1 cells. After 3 days of SiRNA induction, WB analysis showed that the protein production of TNFR1 was significantly inhibited (Figure 5A). ALP staining showed that the expression of ALP increased significantly after TNFR1 knockdown, and adding PGRN further improved the expression of ALP (Figure 5B,C). WB analysis (Figure 5D,E; Figure S2F,G) and real‐time PCR (Figure 5F; Figure S2H,I) for RUNX2 and Col‐1 also showed that the expression of RUNX2 and Col‐1 increased significantly after TNFR1 knockdown and that adding PGRN further improved the expression of RUNX2 and Col‐1. Co‐IP experiments confirmed the interaction between PGRN and TNFR1 during osteoblast activation (Figure 5G). During osteoblast activation, the addition of PGRN significantly reduced the interaction strength between TNFα and TNFR1 (Figure S3C,D). At the same time, we confirmed our findings with BMSC, showing that ALP expression increased after TNFR1 knockdown (Figure 5H,I). PGRN reduced TNFα‐mediated inhibition of osteoblast differentiation by interacting with TNFR1 and PGRN could promote osteoblast differentiation in another way.

### 3.6 PGRN Promoted Osteoblast Differentiation via Interacting With TNFR2

**FIGURE 6:**
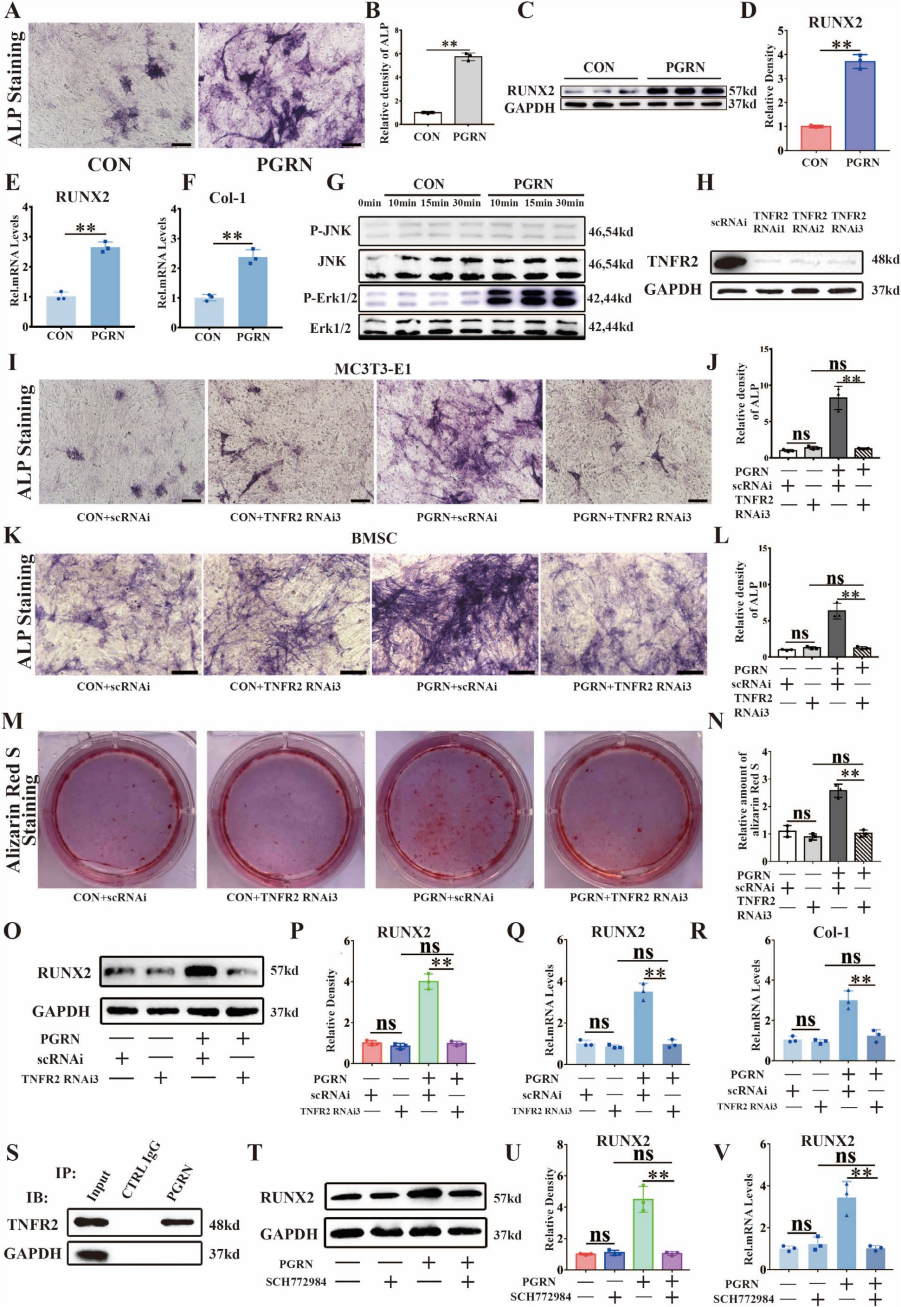
PGRN promoted osteoblast differentiation via interacting with TNFR2. (A) Representative images of ALP staining of MC3T3‐E1 cells co‐cultured with PGRN for osteogenic induction for 14 days. Scale bars, 100 μm. (B) Quantification of ALP staining analysis (*n* = 3 for each group). (C) Western blot analysis of RUNX2 of the indicated groups after cultured for 3 days. (D) Quantification of WB analysis (*n* = 3 for each group). (E, F) Real‐time PCR of RUNX2 of the indicated groups (*n* = 3 for each group) after cultured for 8 h. (G) Western blot (WB) analysis of p‐Erk1/2, Erk1/2, p‐JNK and JNK of MC3T3‐E1 cells cultured with PGRN. (H) WB analysis of TNFR2 to detect the knockdown efficacy of TNFR2 RNAi in MC3T3‐E1 cells after cultured for 3 days. (I) Representative images of ALP staining of MC3T3‐E1 cells co‐cultured with PGRN and TNFR2 RNAi for osteogenic induction for 14 days. Scale bars, 100 μm. (J) Quantification of ALP staining analysis (*n* = 3 for each group). (K) Representative images of ALP staining of BMSCs co‐cultured with PGRN and TNFR2 RNAi for osteogenic induction for 14 days. Scale bars, 100 μm. (L) Quantification of ALP staining analysis (*n* = 3 for each group). (M) Representative images of Alizarin Red S staining of MC3T3‐E1 cells co‐cultured with PGRN and TNFR2 RNAi for osteogenic induction for 21 days. (N) Quantitative analysis of Alizarin Red S staining was detected by relative amounts of Alizarin Red S (*n* = 3 for each group). (O) Western blot analysis of RUNX2 of the indicated groups after stimulated for 3 days. (P) Quantification of WB analysis (*n* = 3 for each group). (Q, R) Real‐time PCR of RUNX2 and Col‐1 of the indicated groups (*n* = 3 for each group) after cultured for 8 h. (S) Immunoprecipitation (IP) with anti‐PGRN or control IgG followed by Western blotting for TNFR2 (*n* = 3). IB: Immunoblot. (T) Western blot analysis of RUNX2, after MC3T3‐E1 cells cultured with SCH772984 (5 μM) and PGRN for 3 days. (U) Quantification of WB analysis (*n* = 3 for each group). (V) Real‐time PCR of RUNX2 of the indicated groups (*n* = 3 for each group) after cultured for 8 h. Concentration: TNFα (10 ng/mL), rhPGRN (500 ng/mL), SCH772984 (5 μM). Data were presented as the mean ± SD. **p < 0.01.

To further clarify whether PGRN can promote osteoblast differentiation, we added PGRN to the osteogenic differentiation medium. As indicated in Figure 6A,B, ALP staining showed that PGRN promoted the expression of ALP. PGRN also promoted the protein production of RUNX2 (Figure 6C,D). Real‐time PCR revealed that PGRN increased the transcription levels of RUNX2 and Col‐1 (Figure 6E,F). In the process of osteogenic differentiation promoted by PGRN, the protein phosphorylation level of Erk1/2 was significantly increased (Figure 6G). To determine whether PGRN promotes osteogenic differentiation through TNFR2, we used specific siRNAs and TNFR2 neutralising antibody to inhibit TNFR2 expression in MC3T3‐E1 cells. WB analysis showed that the protein production of TNFR2 was significantly inhibited (Figure 6H). ALP staining showed that the expression of ALP decreased significantly after TNFR2 knockdown (Figure 6I,J). In BMSC cells, the osteogenic effect of PGRN was inhibited after knockdown of TNFR2 expression (Figure 6K,L). Knockdown of TNFR2 reduced the amount of alizarin red S (Figure 6M,N). Furthermore, WB (Figure 6O,P, Figure S2J,K) analysis for RUNX2 and real‐time PCR (Figure 6Q,R;Figure S2L,M) for RUNX2 and Col‐1 indicated that PGRN could not promote osteogenic differentiation after TNFR2 knockdown. Immunofluorescence staining showed the co‐localization of TNFR2 and PGRN (Figure S4). Co‐IP experiments confirmed the interaction between PGRN and TNFR2 during osteoblast activation (Figure 6S). To further investigate the involvement of the ERK signalling pathway, ERK inhibitor SCH772984 was added at the same time with PGRN. As shown in Figure 6T,U, after blocking the ERK pathway, RUNX2 expression was significantly reduced. Real‐time PCR (Figure 6V) indicated after blocking the ERK pathway, the promotion of RUNX2 by PGRN was inhibited.

## 4 Discussion

Osteoporosis is a metabolic disease characterised by bone loss and microstructure destruction, which often leads to pain and fractures and imposes a heavy burden on society, families and affected patients [44]. Under normal physiological conditions, there is a relatively stable dynamic balance between osteoclasts and osteoblasts. Thus, the root cause of osteoporosis is the disturbance in the bone metabolic balance, specifically, inadequate bone formation mediated by osteoblasts or excessive bone resorption mediated by osteoclasts [1, 2]. Therefore, maintenance of the balance in bone metabolism is the focus of osteoporosis prevention and treatment.

**FIGURE 7:**
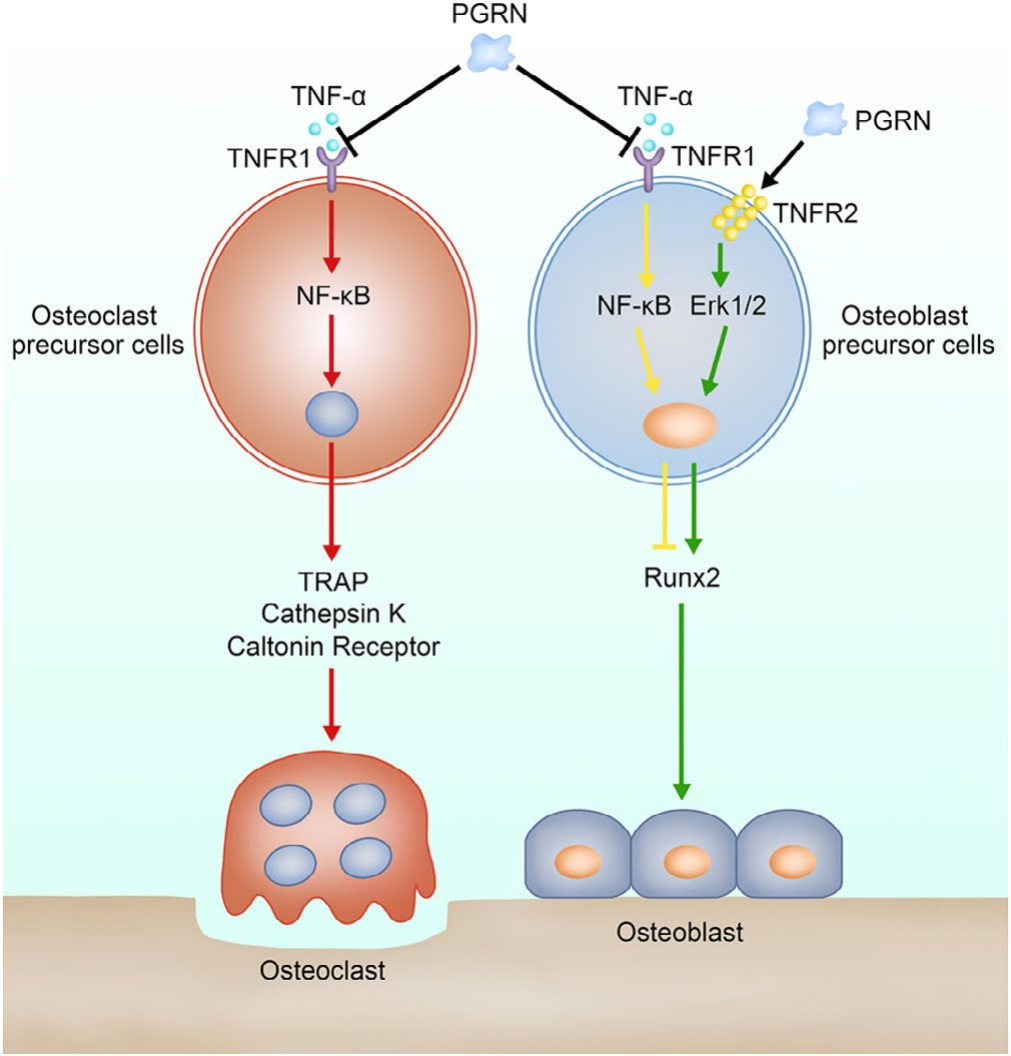
Schematic diagram of functional model of PGRN in osteoporosis.

Recent studies have shown that osteoclast differentiation is mainly regulated by RANKL and macrophage colony‐stimulating factor (M‐CSF) [11]. TNFα has been reported to stimulate osteoclast differentiation through this pathway [45]. Other studies have shown that TNFα can inhibit osteoblast differentiation by inhibiting osteoblast recruitment. In addition, titanium particles have been shown to increase the TNFα concentration locally and promote skull osteolysis by activating the NF‐κB pathway [36]. These studies suggested that TNFα plays an important role in bone metabolic balance. Therefore, TNFα was selected to stimulate osteogenic differentiation or osteoclast differentiation in our experiments. Our results suggested that TNFα affects bone metabolic balance in two main ways. On one hand, TNFα promoted osteoclast differentiation through the NF‐κB and P38 MAPK pathways. On the other hand, TNF inhibited osteoblast differentiation and matrix mineralization through the NF‐κB and P38 MAPK pathways (Figure 7). Since TNFα affects the balance of bone metabolism, suppression of TNFα‐induced inflammatory osteoporosis may be a potential target for the treatment of osteoporosis.

PGRN could effectively interact with tumour necrosis factor receptors (TNFRs) [31]. Current studies indicate that there are two main types of receptors for TNFRs, namely, TNFR1 and TNFR2. TNFR1 has been widely studied and regarded as an important proinflammatory mediator [46]. TNFR2 is currently thought to be involved in processes such as heart failure and cartilage repair. PGRN interacts competitively with TNFα to TNFR1 [47]. For interacting with TNFR2, PGRN shows a much higher interaction than TNFα [48]. Given that PGRN interacts with the same receptor as TNFα, many studies have shown that PGRN can inhibit TNFα‐mediated diseases such as osteoarthritis, intervertebral disc degeneration and colitis [49].

In view of the important role of PGRN in bone metabolism [31, 36, 37, 39], and the close correlation between PGRN and TNFR. We used PGRN to our experiments to study its role in osteoporosis. Our results suggested that PGRN can affect bone metabolism in three ways. First, PGRN inhibits TNFα‐induced osteoclastogenesis by interacting with TNFR1 to reduce the phosphorylation levels of the P65 and P38 proteins. Second, PGRN restores TNFα‐induced inhibition of osteogenic differentiation by interacting with TNFR1 to reduce the phosphorylation levels of the P65 and P38 proteins. Third, PGRN promotes osteogenic differentiation by interacting with TNFR2 to increase the phosphorylation of Erk1/2 (Figure 7). Finally, the addition of PGRN alone can promote osteoblast differentiation, but the addition of PGRN alone has no significant effect on osteoclast differentiation. This is largely due to the fact that the osteoclast differentiation process depends on the activation of the RANKL system, and TNFα can strongly activate the RANKL system. Although PGRN inhibits osteoclast differentiation by interacting with TNFα, PGRN alone has no significant effect on the RANKL system.

However, other authors found that PGRN activates osteoclast differentiation, specifically the formation of multinucleated cells [50]. This difference may be due to the difference in the number of days PGRN acts on cells. PGRN contains seven‐and‐a‐half repeats of a cysteine‐rich motif and can be hydrolyzed to granulin (GRN). GRN has been reported to promote inflammation. In this study, BMM cells were incubated with PGRN for at least 14 days. In the process, PGRN may be hydrolyzed into GRN. Thus, PGRN may promote osteoclast differentiation [51]. The specific cause depends on the ratio of PGRN/ GRN in the medium. In the early stage, PGRN has an intact construction and can fully play its role. While in late stage, amount of PGRN is hydrolyzed into GRN, both of which play their respective roles. So, it seems PGRN has a dual effect on osteoclast differentiation, but we believe PGRN exerts its effect due to the ratio of PGRN/GRN.

In addition, we examined the relationship between three markers of bone metabolism and serum levels of PGRN. Among these, β‐cross had a strong correlation. Current studies have indicated that β‐cross is a product released into the blood after bone tissue is dissolved and absorbed, which reflects the degree of bone absorption [52]. The results of our study showed that PGRN serum content was negatively correlated with β‐cross, which was consistent with our experimental results that PGRN inhibited osteoclast differentiation. Therefore, our results indicated that PGRN was a protective factor in the serum of osteoporosis patients and was negatively correlated with bone resorption.

In conclusion, PGRN attenuates TNFα‐induced osteoporosis by interacting with TNFR1 and promotes osteoblast genesis by interacting with TNFR2. Therefore, PGRN may become a new target for the treatment of osteoporosis and a serum indicator for diagnosis.

## Author Contributions

**Yuanqiang Zhang:** data curation (lead), formal analysis (equal), resources (equal), software (equal). **Shaoyi Wang:** investigation (equal), visualization (equal), writing – original draft (equal), writing – review and editing (equal). **Xiaocong Zhou:** conceptualization (equal), data curation (equal). **Hengyan Zhang:** conceptualization (equal), supervision (equal). **Haoxin Zhai:** supervision (equal), writing – original draft (equal). **Qiting He:** investigation (equal), methodology (equal). **Xuetao Zhu:** data curation (equal). **Yanbin Zhu:** methodology (equal), resources (equal), software (equal).

## Ethics Statement

This study was approved by the Ethics Committee of Animal Medicine in Qilu Hospital of Shandong University (Jinan) and conducted according to the relevant guidelines. (KLYY‐2015‐088).

## Conflicts of Interest

The authors declare no conflicts of interest.

## Supporting information


Data S1.

## Data Availability

The data that support the findings of this study are available on request from the corresponding author. The data that support the findings of this study are also available in the Supporting Information of this article.
