# Supplementary material for: Progranulin Protects Against Osteoporosis by Regulating Osteoclast and Osteoblast Balance via TNFR Pathway
Source: J Cell Mol Med. 2025 Feb 5;29(3):e70385. doi: 10.1111/jcmm.70385 (PMC11798871; doi:10.1111/jcmm.70385)
Supplement: Supplementary file 1 — Data S1. [file JCMM-29-e70385-s001.docx]

**Progranulin protects against osteoporosis by regulating Osteoclast and Osteoblast balance via TNFR pathway**

Shaoyi Wang^1,2,3^, Yanbin Zhu^4^, Zihao Wang^1,2^, Haoxin Zhai^1,2^, Qiting He^1,2^, Xuetao Zhu^1,2^, Yuanqiang Zhang^1, #^

^1^ Department of Orthopaedic Surgery, Qilu Hospital of Shandong University, Jinan, Shandong, P. R. China.

^2^ Cheeloo College of Medicine, Shandong University, Jinan, Shandong, P. R. China.

^3^ Laboratory of Basic Medical Sciences, Qilu Hospital, Cheeloo College of Medicine, Shandong University, Jinan, Shandong, P. R. China.

^4^ Department of Orthopaedic Surgery, The Third Hospital of Hebei Medical University, Shijiazhuang, P. R. China.

#Corresponding author address: Shandong University Qilu Hospital, No.107, Wen Hua Xi Road, Jinan, Shandong, 250012, P.R. China.

Yuanqiang Zhang: yqzhangQLYY@126.com

**Materials and Methods**

**Immunofluorescence staining (IF)**

MC3T3-E1 cells were incubated with rhPGRN for 1hours and then fixed either immediately or after 24 hours using 4% paraformaldehyde. Permeabilization was performed with 0.2% Triton-X 100 for 20 minutes, followed by blocking with 1% bovine serum albumin (BSA) for 30 minutes. Subsequently, the cells were incubated overnight at 4°C with primary antibodies targeting various proteins, including PGRN (1: 1500, Abcam, USA) and rabbit-anti-TNFR2(1:1000, Affinity, USA). The following day, the cells were incubated for 1 hour with fluorescently conjugated goat anti-rabbit IgG secondary antibodies (1:100, Abbkine, China). Imaging was performed using an IX71-SIF fluorescence microscope (Olympus, Tokyo, Japan), and the data were analyzed with Image-Pro Plus 6.0 software (Media Cybernetics, Inc., USA).

**Treatment with TNFR1 and TNFR2 neutralizing antibody**

During osteoclast or osteoblast differentiation, BMMs, MC3T3-E1 cells and BMSCs were incubated with 10 ng/ml TNFα with or without 500 ng/ml rhPGRN and 20ug/ml TNFR1 neutralizing antibody (R&D Systems, USA). During osteoblast differentiation, MC3T3-E1 cells and BMSCs were incubated with 500 ng/ml rhPGRN with or without 20ug/ml TNFR2 neutralizing antibody (R&D Systems, USA)[1]. Cells were harvested and tested at 8 hours by real-time PCR, at 3 days by Western blotting.

**RNA Extraction and Real-Time PCR**

Total RNA was extracted with TRIzol reagent (Takara Bio, Japan) after cell treatment for 8 h. Total RNA (1 µg) was reverse-transcribed to complementary DNA (cDNA) using a cDNA Synthesis Kit (Toyobo, Japan) and used with SYBR Green PCR Matrix Mix (Toyobo, Japan) for real-time PCR. The reverse transcription parameter is set to: 37 °C for 15 min, 98 °C for 5 min. The cycle parameters of Real-time PCR were set to: 95 °C for 1 min, 40 cycles (95 °C for 15 s, 60 °C for 15s, and 72 °C for 45 s) and 72 °C for 5 min. Sequence-specific primers for PGRN, TRAP, cathepsin K, calcitonin receptor, RUNX2, and Col-1 are listed in Table 2. The difference between groups was compared by 2^-∆∆Ct^.

**TRAP staining**

BMMs were seeded into 6-well plates at a suitable density. After 7 days of stimulation, the cells were washed with PBS and fixed with 4% paraformaldehyde. TRAP staining followed the instructions of the TRAP staining kit (P0332, Beyotime, China)[2]. Wine red multinucleated cells are considered TRAP+ cells. Ten fields were randomly selected and the total number of TRAP+ cells was counted. Image results were analyzed by ImageJ Pro. The number of TRAP+ cells in each group was used to measure differences between groups.

**ALP and Alizarin red staining**

MC3T3-E1 cells and BMSCs were seeded into 6-well plates at a suitable density. After two weeks of stimulation, a naphthol phosphate substrate and fast violet B were used to detect the ALP product (C3206, Beyotime, China)[3]. After three weeks of stimulation, alizarin red S staining was used to evaluate calcium deposition (C0148S, Beyotime, China)[4]. Two secondary wells were used for each set of experiments. Image results were analyzed by ImageJ Pro. The difference between groups was determined by comparing the relative IOD of ALP staining and the number of calcium nodules.

**Knockdown of TNFR1 and TNFR2 by siRNA**

To knock down TNFR1 and TNFR2, BMMs, MC3T3-E1 cells and BMSCs were transfected with siTNFR1, siTNFR2, and scrambled control siRNA (scRNAi) by using the siRNA transfection reagent PROTPCOL (Polyplus, France). Transfection efficiency was assessed by Western blotting 3 days later. After incubation with siTNFR1, siTNFR2 or scRNAi for 3 days, osteoblast or osteoclast differentiation was performed.

**Bone metabolism marker series**

Bone metabolism indexes detected in this study were Total type 1 collagen amino-terminal prolongation peptide (T-P1NP), β-Collagen specific sequence (β-cross) and Vitamin D (VIT-D). Serum samples were tested by Roche electrochemiluminescence automatic immunoassay (cobas e 411, ROCHE, Switzerland) for bone metabolism.

**Enzyme-linked immunosorbent assay (ELISA)**

The serum concentration of progranulin (PGRN) was measured using a Human PGRN ELISA Kit (Abcam, USA) according to the manufacturer’s instructions. The optical density at 450 nm was assayed by a Varioskan flash multifunction plate reader (Thermo Fisher Scientific, Waltham, MA). Cytokine concentrations (pg/mL or ng/ml) were computed with a standard reference curve generated by CELLQUEST software.


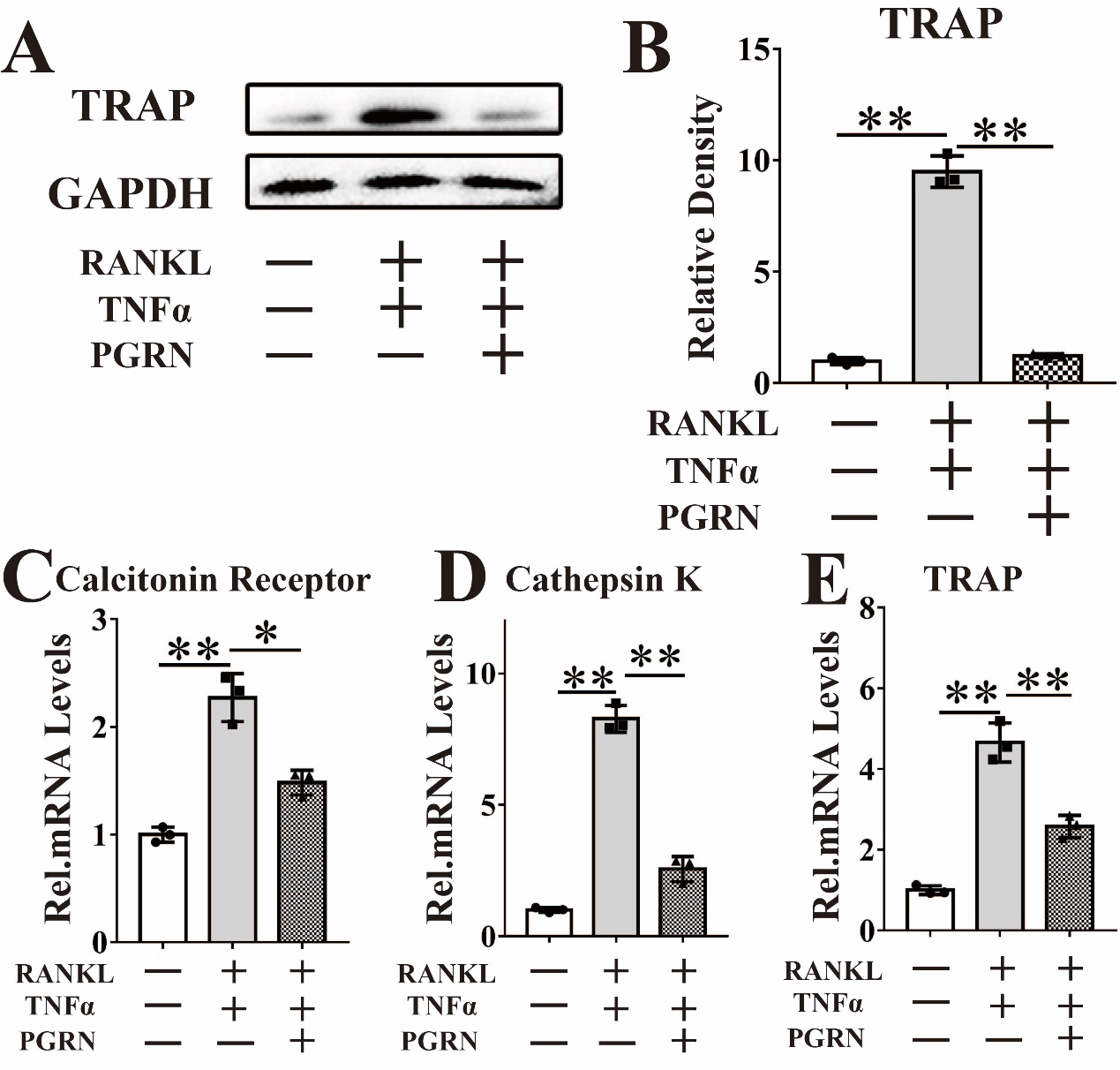


**Figure S1. PGRN suppressed TNFα induced inflammatory osteoclast differentiation**

(A). Western blot (WB) analysis of TRAP of RAW 264.7 cells after incubated with TNFα and PGRN for 3 days. (B). Quantification of WB analysis (n=3 for each group). (C-E). Real-time PCR of Calcitonin Receptor, Cathepsin K and TRAP after addition of TNFα and PGRN (n=3 for each group). Data were presented as the mean ± SD. *P<0.05, **P<0.01.


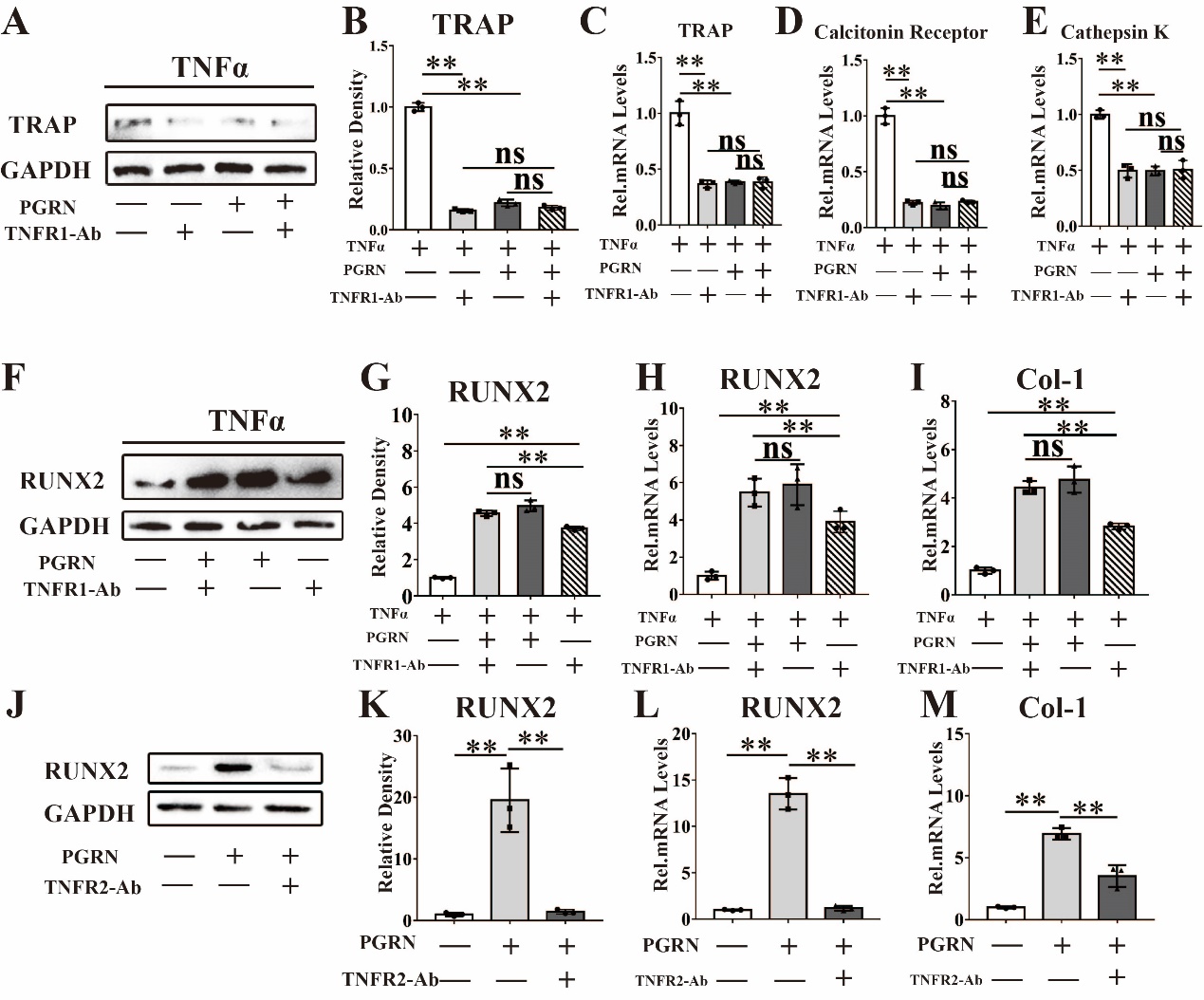


**Figure S2. PGRN inhibits the role of TNFα in osteoclast and osteoblast differentiation through TNFR1 and promotes osteoblast differentiation through TNFR2**

(A). Western blot (WB) analysis of TRAP of BMMs after incubated with TNFα, PGRN and TNFR1-Ab for 3days. (B). Quantification of WB analysis (n=3 for each group). (C-E). Real-time PCR of Calcitonin Receptor, Cathepsin K and TRAP after addition of TNFα, PGRN and TNFR1-Ab (n=3 for each group). (F). Western blot (WB) analysis of RUNX2 of MC3T3-E1cells after incubated with TNFα, PGRN and TNFR1-Ab for 3 days. (G). Quantification of WB analysis (n=3 for each group). (H-I). Real-time PCR of RUNX2 and Col-1 after addition of TNFα, PGRN and TNFR1-Ab (n=3 for each group). (J). Western blot (WB) analysis of RUNX2 of MC3T3-E1 cells after incubated with PGRN and TNFR2-Ab for 3 days. (K). Quantification of WB analysis (n=3 for each group). (L-M). Real-time PCR of RUNX2 and Col-1 after addition of PGRN and TNFR2-Ab (n=3 for each group). Data were presented as the mean ± SD. *P<0.05, **P<0.01.


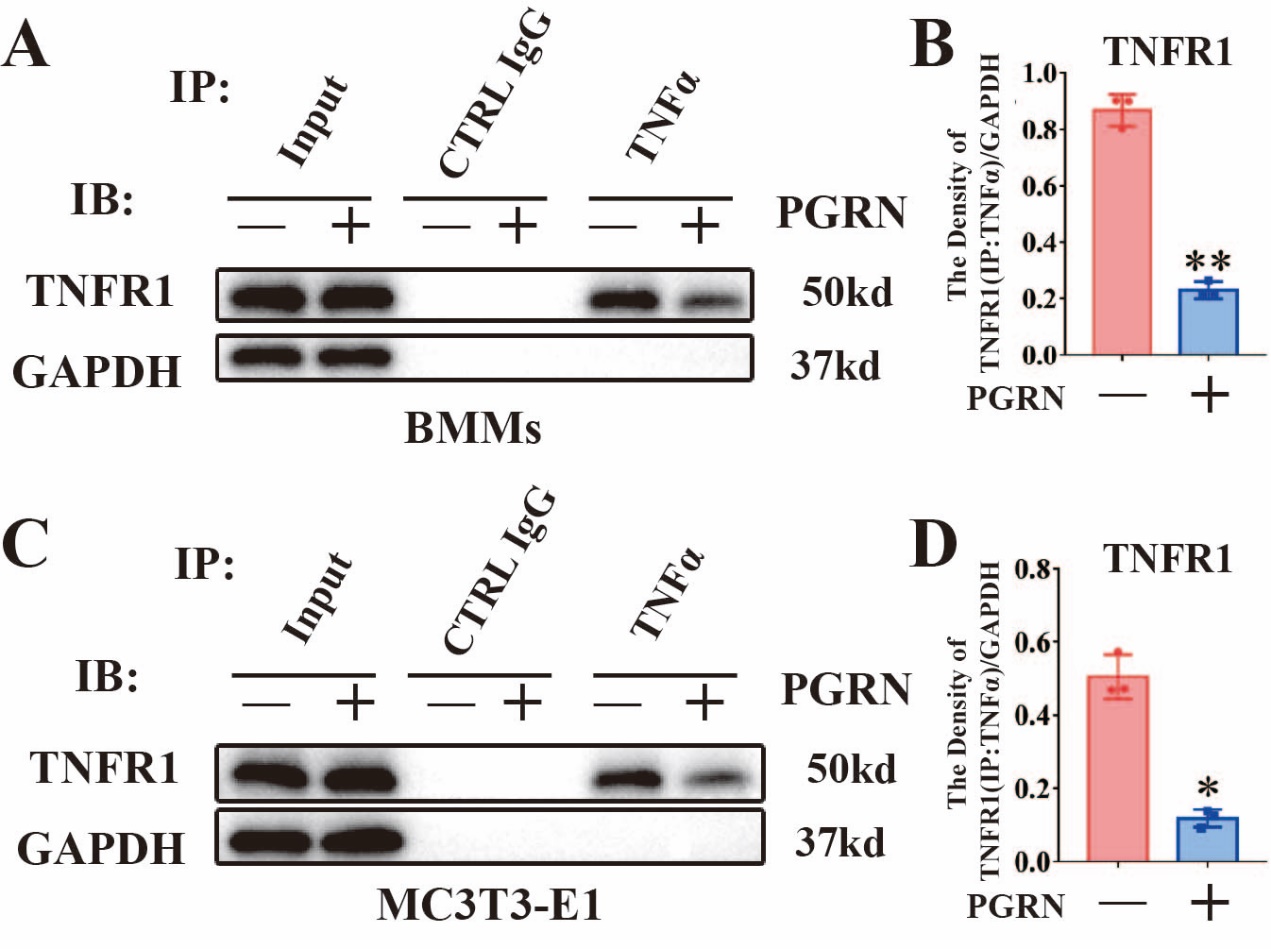


**Figure S3. PGRN reduced the strength of the interaction between TNFR1 and TNFα on osteoclast precursors cell and osteoblast precursors cell.**

(A). Immunoprecipitation (IP) with anti-TNFα or control immunoglobulin G (IgG) followed by Western blotting for TNFR1 (n = 3) on BMMs. IB: immunoblot. (B). The density of TNFR1(IP: TNFα)/GAPDH was used to analyzed the interaction between TNFR1 and TNFα (n=3 for each group). (C). IP with with anti-TNFα or control immunoglobulin G (IgG) followed by Western blotting for TNFR1 (n = 3) on MC3T3-E1 cells. (D). The density of TNFR1(IP: TNFα)/GAPDH was used to analyzed the interaction between TNFR1 and TNFα (n=3 for each group). Concentration: TNFα (10 ng/ml), rhPGRN (500 ng/ml). Data were presented as the mean ± SD. *P<0.05, **P<0.01.


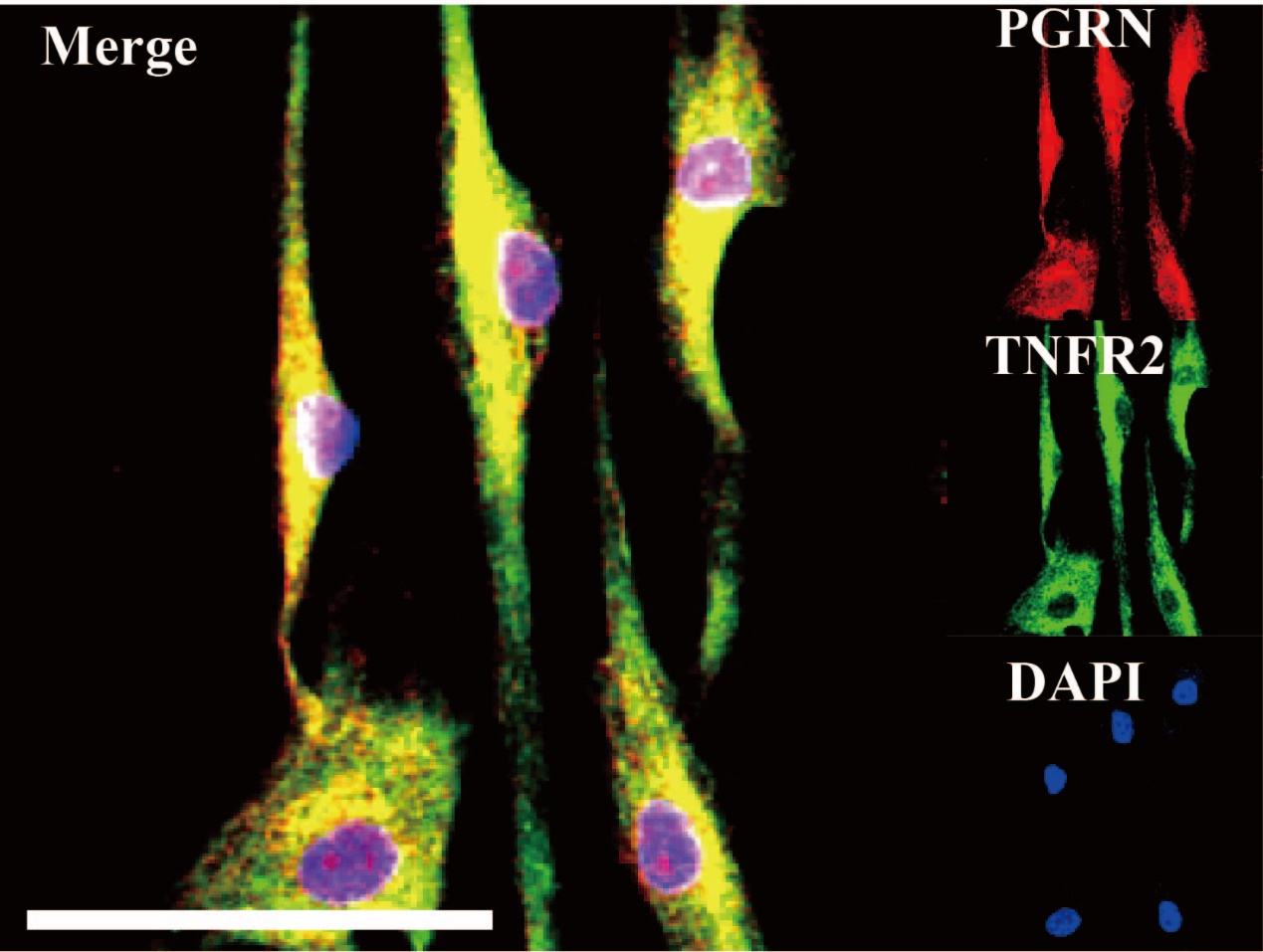
**Figure S4 Representative immunostaining of TNFR2 showing colocalization with PGRN (n = 3). Scale bars, 100 μm.**

**Reference:**

1. R. Gordon, V. Anantharam, A. Kanthasamy, and A. Kanthasamy, *Proteolytic activation of proapoptotic kinase protein kinase Cδ by tumor necrosis factor α death receptor signaling in dopaminergic neurons during neuroinflammation.* Journal of neuroinflammation, (2012). 9: p. 82.doi:10.1186/1742-2094-9-82

2. J. Lin, H. Wu, Y. Liu, P. Shaw, and P. Li, *N16 suppresses RANKL-mediated osteoclatogenesis by down-regulating RANK expression.* International journal of biological macromolecules, (2020). 151: p. 1154-1162.doi:10.1016/j.ijbiomac.2019.10.159

3. Z. Yin, X. Chen, J. Chen, W. Shen, T. Hieu Nguyen, L. Gao, et al., *The regulation of tendon stem cell differentiation by the alignment of nanofibers.* Biomaterials, (2010). 31(8): p. 2163-75.doi:10.1016/j.biomaterials.2009.11.083

4. H. Chi, M. Kong, G. Jiao, W. Wu, H. Zhou, L. Chen, et al., *The role of orthosilicic acid-induced autophagy on promoting differentiation and mineralization of osteoblastic cells.* Journal of biomaterials applications, (2019). 34(1): p. 94-103.doi:10.1177/0885328219837700
